# Using Gini coefficient to determining optimal cluster reporting sizes for spatial scan statistics

**DOI:** 10.1186/s12942-016-0056-6

**Published:** 2016-08-03

**Authors:** Junhee Han, Li Zhu, Martin Kulldorff, Scott Hostovich, David G. Stinchcomb, Zaria Tatalovich, Denise Riedel Lewis, Eric J. Feuer

**Affiliations:** 1 Division of Biostatistics, Research Institute of Convergence for Biomedical Science and Technology, Pusan National University Yangsan Hospital, Pusan, Korea; 2 Surveillance Research Program, Division of Cancer Control and Population Sciences, National Cancer Institute, National Institutes of Health, Bethesda, MD 20892 USA; 3 Brigham and Women’s Hospital and Harvard Medical School, Boston, MA USA; 4 Information Management Services, Calverton, MD USA; 5 Westat, Inc, Rockville, MD USA

**Keywords:** Scan statistic, SaTScan, Cluster detection, Cancer mortality, Log likelihood ratio, Cluster reporting size, Gini coefficient, Spatial statistics, Disease surveillance

## Abstract

**Background:**

Spatial and space–time scan statistics are widely used in disease surveillance to identify geographical areas of elevated disease risk and for the early detection of disease outbreaks. With a scan statistic, a scanning window of variable location and size moves across the map to evaluate thousands of overlapping windows as potential clusters, adjusting for the multiple testing. Almost always, the method will find many very similar overlapping clusters, and it is not useful to report all of them. This paper proposes to use the Gini coefficient to help select which of the many overlapping clusters to report.

**Methods:**

The Gini coefficient provides a quick and intuitive way to evaluate the degree of the heterogeneity of the collection of clusters, which is useful to explain how well the cluster collection reveal the underlying true cluster patterns. Using simulation studies and real cancer mortality data, it is compared with the traditional approach for reporting non-overlapping clusters.

**Results:**

The Gini coefficient can identify a more refined collection of non-overlapping clusters to report. For example, it is able to determine when it makes more sense to report a collection of smaller non-overlapping clusters versus a single large cluster containing all of them. It also fulfils a set of desirable theoretical properties, such as being invariant under a uniform multiplication of the population numbers by the same constant.

**Conclusions:**

The Gini coefficient can be used to determine which set of non-overlapping clusters to report. It has been implemented in the free SaTScan™ software version 9.3 (www.satscan.org).

## Background

Spatial and spatio-temporal scan statistics play an increasingly important role in public health surveillance. Cluster-detection tools based on these statistics have been broadly utilized in identifying geographic patterns and clusters of chronic diseases [1], detecting outbreaks of communicable diseases [2, 3], as well as linking possible risk factors to disease outcomes [4]. A likelihood-based approach allows the scan statistic to identify clusters and evaluate if they are statistically significant, adjusting for the multiple testing inherent in the many potential cluster locations and sizes.

A variable sized candidate area (scanning window) literally scans across the study region. For each window, the likelihood is calculated and the candidate area with the maximum likelihood defines the most likely cluster. Several methods have been proposed in this class. This includes circular and elliptic spatial scan statistics [5, 6], as well as non-parametric spatial scan statistics that aim to detect irregular shaped cluster. The latter are non-parametric in terms of the spatial cluster shapes, not in terms of the likelihood functions, and they include e.g. Duczmal and Assunção’s [7] simulated annealing scan statistic, Tango’s [8] flexibly shaped spatial scan statistic, Patil and Taillie’s [9] upper-level set scan statistic, Gangnon and Clayton’s [10, 11] likelihood-based method, Costa et al.’s [12, 13] spanning tree scan statistics, and Duczmal et al.’s [14] genetic algorithm scan statistic.

Many studies have been conducted on the statistical power of scan statistics with predefined varying shapes and sizes. As one would intuitively expect, the circular spatial scan statistic has best power for compact clusters while non-parametric scan statistics have best power for irregularly shaped clusters [15–19]. Specifically, Huang et al. [17] performed an intensive simulation study on power and sample size requirements for tests with different spatial patterns in terms of geographic locations and relative risks. They found that the elliptic version performs well for cluster detection in data with a variety of spatial patterns. Based on their findings, we focus on the elliptic purely spatial scan statistic but the principles and theory described here applies to other likelihood-based methods and the space–time scan statistics as well.

The spatial scan statistic requires a maximum spatial window size (MSWS). In the SaTScan™ software, this can be defined based on the size of the population or the geographic area of the study region. The maximum spatial window size is often defined to be less than or equal to 50 % of the total population at risk (Table 1). Ribeiro and Costa [20, 21] explored different values for the maximum cluster size and suggested that performance is sensitive to the maximum cluster size chosen by the user. This is natural. With a larger maximum, a much larger set of potential clusters are evaluated, so there is more multiple testing to adjust for. More importantly, with a smaller maximum, larger clusters are not evaluated and cannot therefore be found. A general guideline is to select the maximum so that any clusters larger than that maximum is of no public health interest. While it has sometimes been done (Table 1), one should never run the analysis multiple times using different values for the maximum. If that is done, then those analyses performed with a smaller maximum will not adequately adjust for the multiple testing that was done when also evaluating the larger clusters. If multiple maxima were used by mistake, one should report the p values from the largest maximum used.

**Table 1:** Selection of maximum spatial window size in 81 recent publications

Maximum spatial window size	<5 %	10 %	15 %	25 %	30 %	50 %	Multiple maxima	Distance based	Not specified	Total
# of pub.	5	3	1	5	1	22	8	13	23	81
% of pub.	6	4	1	6	1	27	10	16	28	100

Using Google Scholar, we ran a search of publications with both words “SaTScan” and “cancer” published during 2015 and yielded 156 results. Restricting the search to scientific papers published in peer-reviewed journals in English, we found a total of 81 papers using the SaTScan™ software (www.satscan.org). This table summarizes the maximum spatial window size (MSWS) used in these 81 papers. 8 papers (10 %) erroneously (see reason in “Maximum spatial window size of reported clusters” section) used multiple MSWS ranging from 2 to 4 choices, with 50 % always included as one of them

When rejecting the null hypothesis in a spatial scan statistic analysis, there are almost always multiple statistically significant clusters that overlap each other and the number can be in the thousands. While the SaTScan software can provide all of these overlapping clusters, it is not meaningful to report all of them, since many of them are almost identical. As the default, SaTScan has reported clusters hierarchically, first reporting the cluster with the maximum likelihood, and then reporting the one with the maximum likelihood among the remaining clusters that do not overlap an already reported cluster. When using a large maximum window size, the maximum likelihood will sometimes be obtained for a large cluster that contains several smaller clusters, and it is not always clear whether it is better to report one large cluster or several smaller ones. The decision on which collection of clusters better represents the underlying patterns is currently very subjective due to a lack of systematic ways to evaluate the cluster models. Specifically, as we will see later, the hierarchical approach may turn up with unnecessarily large and less informative clusters. Within a pre-determined maximum cluster size such as 50 % of the population at risk, the goal of the Gini coefficient, which we propose in this paper, is to determine the best collection of non-overlapping statistically significant clusters to report, from among the many thousands of statistically significant and highly overlapping clusters that the spatial scan statistic finds.

### Most likely cluster and statistical inference

Suppose we have a geographical region partitioned into sub-regions such as counties or census tracts. The areas are represented either by their geometrical or population weighted centroids using the longitude and latitude coordinates, their age and gender specific population at risk, and the number of cancer cases in each area. Centered at each centroid, the spatial scan statistic uses a very large collection of overlapping circles of continuously varying radius to define a set of potential clusters. Alternatively, the method uses a collection of ellipses with continuously varying lengths and widths for a predefined set of shapes and directional angles. An MSWS is defined in terms of geographical area or as a percentage of total population at risk. To define a cluster, the maximum is never chosen as more than 50 % of the total population at risk.

Let Z be the collection of all the geographic units in study region S. Zone *z* consists of neighboring geographic units (e.g. counties) and can have varying shapes and sizes. Let *c*_*z*_ and *n*_*z*_ be the observed number of cases and the expected number of cases (or population) in zone *z*, respectively. Then $C = \sum\nolimits_{z} {c_{z} }$ and $N = \sum\nolimits_{z} {n_{z} }$ will be the total number of cases and the total number of expected cases in *S*. For cancer incidence and mortality, a Poisson model is typically chosen. The likelihood ratio [5] of a zone *z* is then given by

1 $$ LR(z) = \left\{ {\left( {\frac{{c_{z} }}{{n_{z} }}} \right)^{{c_{z} }} } \right.\left. {\left( {\frac{{C - c_{z} }}{{N - n_{z} }}} \right)^{{C - c_{z} }} } \right\} I(c_{z} > n_{z} ) $$

If there is interest in scanning for ‘negative clusters’ with a lower rate than expected, the indicator function is replaced by *I*(*c*_*z*_ < *n*_*z*_) and if the interest is in clusters of both higher and lower rates, the indicator function is removed. It is equivalent but numerically easier to work with the logarithm, and the test statistic is $T = \max_{z} \log (LR(z)) = \max_{z} LLR(z)$. That is, the most likely cluster is the scanning window *z* ∈ *Z*, which maximizes the log likelihood ratio.

The test statistics T follows approximately an extreme value distribution [22, 23], but the exact distribution is unknown, so statistical significance is evaluated using Monte Carlo hypothesis testing. This is done by creating a large number of random data sets generated under the null hypothesis that there is no cluster, and calculating the value of the test statistic for each of those random data sets. The Monte Carlo p value is then calculated as *r*/(1 + *m*) where *r* is the rank of the test statistic from the real data set among all the random data sets and *m* is the number of random data sets. For example, with a statistical significance level of α = 0.05, the cluster will cause a rejection of the null hypothesis if its likelihood ratio is within the highest 5 % among all the maximum likelihood ratio from the one real and the *m* random data sets.

Calculations were performed using SaTScan™ version 9.3.

### Secondary clusters

In addition to the most likely cluster, it is also of interest to know if there are additional clusters present in the data. The secondary clusters in the real data are also compared to the most likely clusters in the random data sets. In this way, they are only statistically significant if they can reject the null hypothesis on their own strength, irrespective of whether the more likely clusters are true clusters or not. Most secondary clusters overlap with and some only differ slightly with a more likely cluster. While these secondary clusters are always evaluated as part of an analysis, it is not meaningful to report all of them. The SaTScan software has several options on how to report overlapping clusters. In version 9.1 and earlier, the default was a hierarchical option of only reporting clusters that do not overlap with an already reported more likely cluster. A consequence of this is that a large most likely cluster can hide several smaller distinct clusters, and the hierarchical approach is not necessarily the best way to select a set of non-overlapping clusters to report.

### Maximum spatial window size of reported clusters

Since the spatial scan statistic evaluates clusters of different window sizes, it is critically important to adjust for the multiple testing generated by all the different window sizes considered. This means that it is incorrect to run the scan statistic multiple times with different values for the MSWS, and then select the clusters with the lowest *p* value. When doing so, one does not properly adjust for all the multiple testing conducted, and the p values will be biased. It is not always the most likely cluster that is of primary importance though, and a set of smaller sub-clusters can sometimes be of greater interest. It is perfectly fine to report only those and their corresponding p values, as long as proper multiple testing adjustment is made for all the smaller and larger clusters that were also evaluated in the analysis. One way to do this in the SaTScan software is to rerun the analysis and request that it only report clusters of a certain maximum size, while still adjusting for the multiple testing inherent in all the sizes considered in the other prior analyses of the same data, by keeping the MSWS fixed at a larger value. This is an advanced feature in the SaTScan software, and multiple analyses can be done repeatedly on the same data with the same fixed MSWS (e.g. 50 %) but with different maximum reported cluster sizes (MRCS, e.g. 5, 10, 20, and 50 %).

**Fig. 1:**
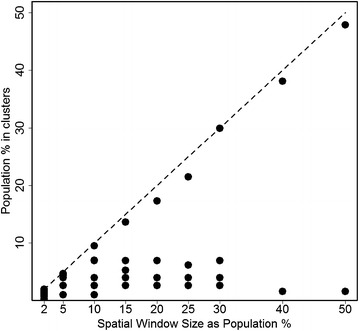
Sizes of clusters by the maximum spatial window size (U.S. Female Lung Cancer Mortality, 2006)

**Fig. 2:**
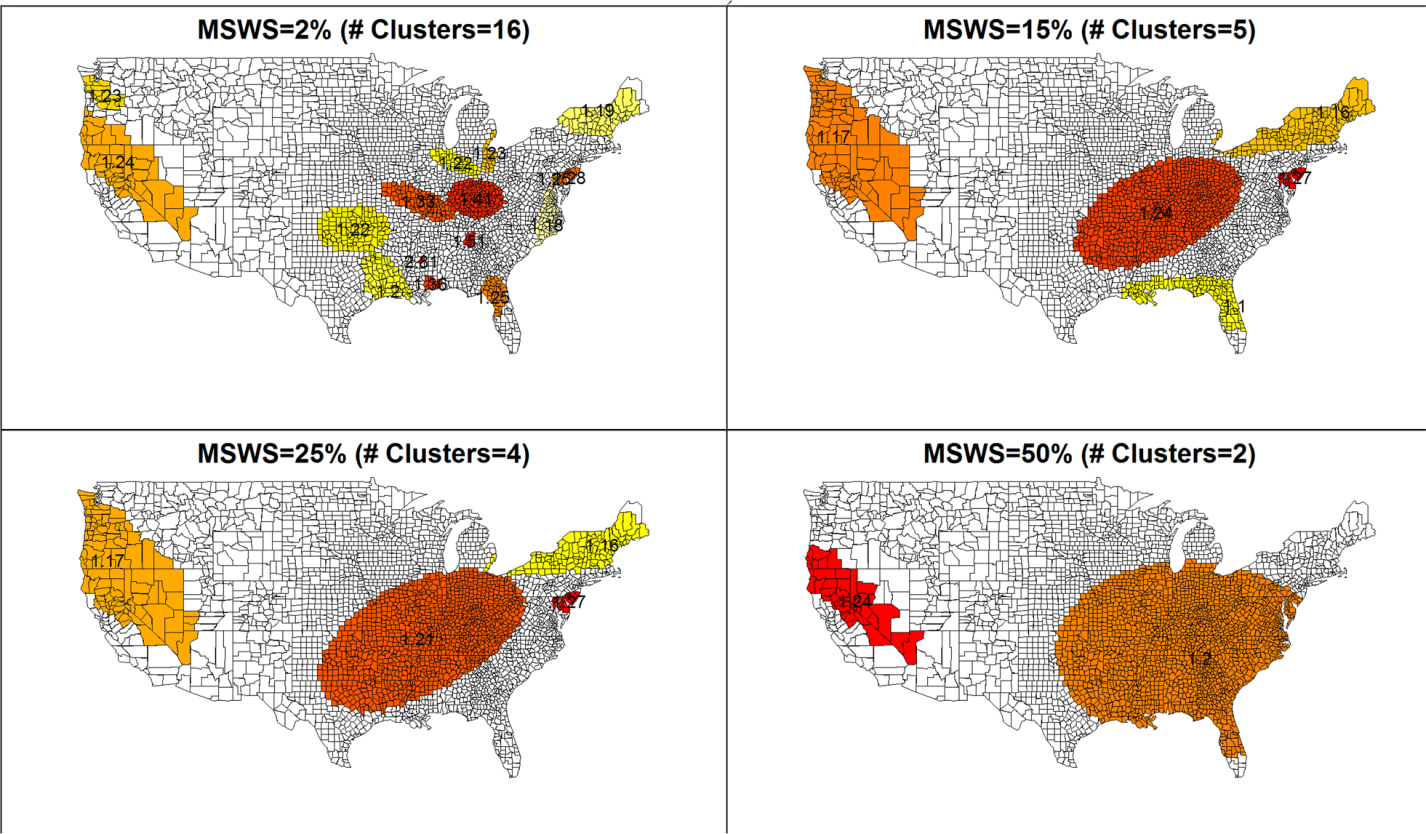
Spatial clusters and the relative risks at various maximum spatial window sizes (U.S. Female Lung Cancer Mortality, 2006; Clusters are identified in *colour* with relative risks labelled on clusters.)

As the sizes of reportable clusters increases, SaTScan often reports a bigger cluster with a size close to the MRCS rather than clusters of smaller or medium size. This is because two or three small clusters close to each other may have a larger likelihood when combined into one big cluster even if there are few observed cases in between the clusters. An example is given in Figs. 1 and 2 where the 2006 U.S. female lung cancer mortality data are used to illustrate the phenomenon. The cancer mortality data were provided by the National Vital Statistics System [24] and accessed through the SEER*Stat software [25]. In both Figures, SaTScan is run with the MRCS at various levels expressed as percentage in the population. Under each user-provided MRCS, clusters are reported in SaTScan and presented here. Figure 1 shows the MRCS (expressed as a percentage in population) as the horizontal axis. The vertical axis represents the percentage of population in each reported cluster, over the total U.S. female population in 2006. The dashed line is the reference that the percentage of cluster population equals to the MRCS. Each dot represents a cluster detected at the corresponding MRCS. Figure 2 selects four levels of MRCS, i.e., MRCS = 2, 15, 25, and 50 % of population, and maps the clusters detected at the corresponding MRCS. Each cluster is illustrated with a difference colour and the relative risk (RR) is labelled on the cluster. When the MRCS is restricted at 2 % of population, SaTScan detects a total of 16 clusters with RR ranging between 1.18 and 2.81 (Fig. 2). Every cluster has a population below the level of MRCS, i.e. 2 % of the total population (Fig. 1). When the MRCS increases, smaller clusters merge into larger ones and the RR’s in the larger clusters tend to be smaller as more and more areas are merged into them. When the MRCS is set at the SaTScan default of 50 % of population, a cluster that accounts for 47 % of U.S. female population is detected with RR of 1.2, along with a small cluster in the northwest of U.S. with RR of 1.24. Figure 1 also shows that at each level of the MRCS, SaTScan always reports a large cluster with the percentage of population very close to the level of MRCS, and the smaller clusters are simply merged into larger ones when the MRCS increases. This observation is true not only for female lung cancer mortality but also for many other cancer sites that we examined, including female breast cancer, prostate cancer, ovary cancer, bladder cancer, and cervical cancer (data not shown), although obviously not for all data sets.

With the Gini coefficient, we will try to find a suitable and informative collection of non-overlapping clusters to report, avoiding overly large clusters with relatively small RR (such as the cluster with 47 % of population and RR = 1.2), as well as many tiny clusters that may blur the big picture of the geographic pattern. The proposed methods in this paper will help to identify clusters with meaningful RR as well as distinct geographic distribution pattern.

An important question is whether there is more evidence for a few larger clusters or several smaller ones. Another question is which clusters to present in a public health or epidemiological research study, if we only want to report non-overlapping clusters. In this paper, we propose a criterion for selecting a set of non-overlapping clusters to report based on the Gini coefficient. Findings from both simulated data and real cancer mortality data show that the Gini coefficient is able to determine when it makes more sense to report a collection of smaller non-overlapping clusters versus a single large cluster containing all of them. It will sometimes avoid overly large clusters with relatively small relative risk (RR), such as the cluster with 47 % of population and RR = 1.2, in favour of a few smaller clusters with higher RR. Compared to using a small MRCS, it will sometimes avoid reporting many tiny clusters that may blur the big picture of the geographic pattern.

## Methods

### Lorenz curve and Gini coefficient

**Fig. 3:**
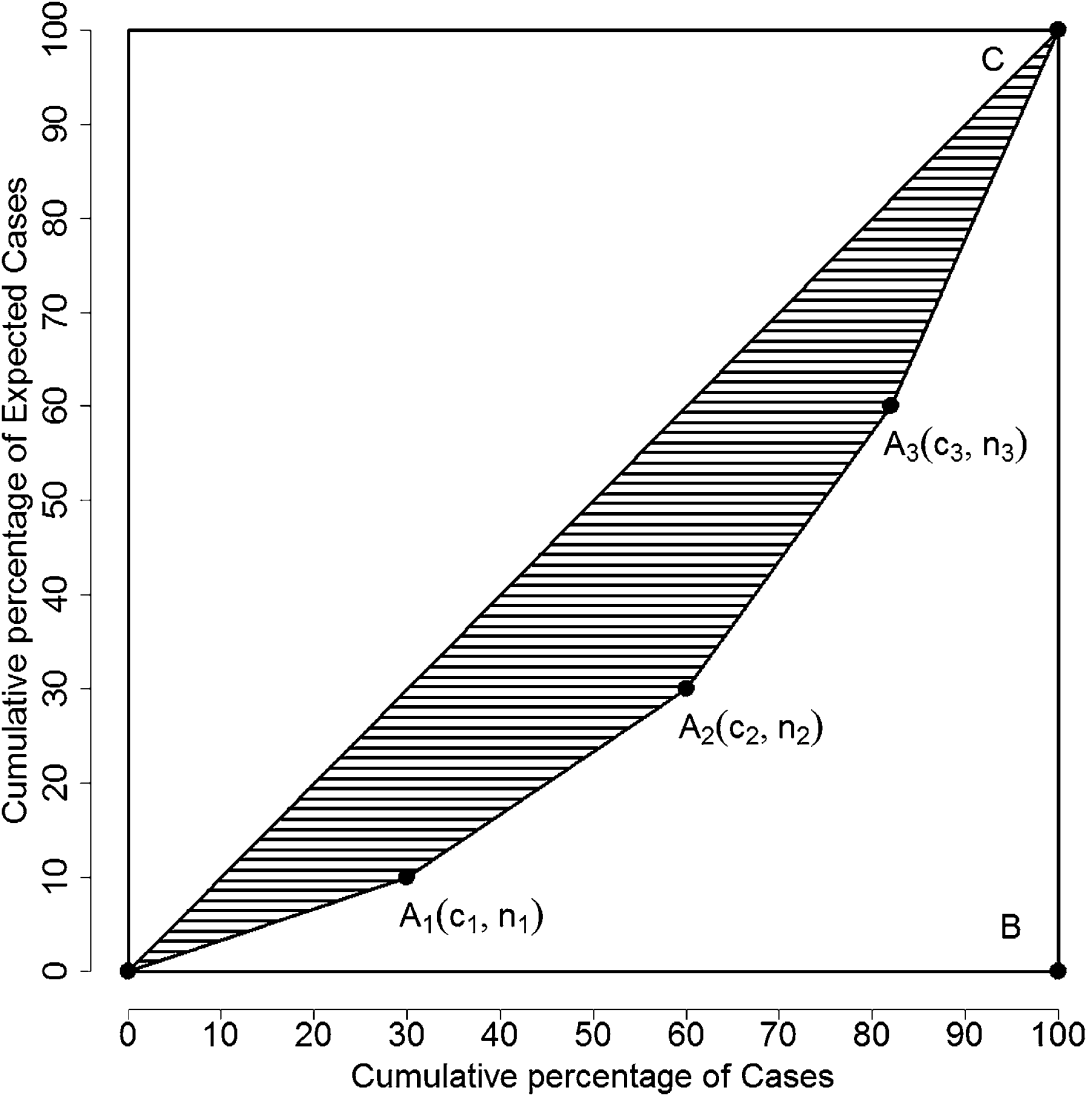
Illustration of Lorentz curve and Gini coefficient for a cluster model with three clusters

In economics, the Lorenz curve [26] is often used to explain and measure the heterogeneity of the wealth distribution. It is a graphical representation of the cumulative distribution function of the empirical probability distribution. The basic format of the graph is a square divided into two symmetric isosceles right triangles (as illustrated in Fig. 3). In this illustration, point *A*_*i*_(*c*_*i*_, *n*_*i*_) denotes that the bottom *n*_*i*_ % of population own *c*_*i*_ % of total wealth and point *A*_*i*_’s are ordered so that the *n*_*i*_ are in non-decreasing order. Line *OC* in the triangle (line *y* = *x*) depicts a perfectly equal wealth distribution and is used as the reference line. The two legs of the triangle, *OB* and *BC*, depict the perfectly unequal wealth distribution, i.e., no household owns any wealth except for the very last one which owns all the wealth in the whole society. Curve *OA*_1_*A*_2_*A*_3_*C* inside the triangle is called the Lorenz curve and describes the observed wealth distribution. The Lorenz curve is then compared to the reference line *OC*, the line for the perfectly equal wealth distribution. The Gini coefficient [27, 28] is a common measure to describe the behaviour of the Lorenz curve. It is simply the ratio of the areas *OA*_1_*A*_2_*A*_3_*C* and *OBC*. Since area *OBC* accounts for ½ of the whole square, the denominator in the Gini coefficient is often taken as ½, hence the value of the Gini coefficient of a Lorenz curve is two times the shaded area. The Lorenz curve and Gini coefficient were originally developed to measure wealth inequality, and have been extended in the area of health disparity in the recent decade. [29–31].

Here we apply the methods of the Lorenz curve and the Gini coefficient to describe collections of disease clusters. If there is a significant cluster in the study region, then the distribution of disease cases tends to be concentrated in the cluster, pushing the Lorenz curve further away from the reference line, and the value of the Gini coefficient will be higher. In Fig. 3, the cumulative percentages of expected cases are on the x-axis and the cumulative percentages of diseases (for example, new cases from cancer) are on the y-axis. The reference line is presenting a perfect equality (or randomness) in the distribution of the deaths, when the cumulative percentages of new cases are exactly the same as the cumulative percentages of the expected cases, i.e. there are no statistically significant clusters. When comparing several competing collections of non-overlapping clusters, the one with the highest Gini coefficient value should be chosen as the cluster collection to report.

In the illustration of Fig. 3, the *x*- and *y*-axes represent the cumulative percentages of observed cases and expected cases, respectively. Each cluster *A*_*i*_ has coordinates ($c_{i}$, $n_{i}$). The origin point *O*’s coordinates are (0, 0) and point *C* has coordinates ($c_{I + 1}$, $n_{I + 1}$) = (1, 1). The relative risk for cluster *A*_*i*_ is calculated as $\frac{{c_{i} - c_{i - 1} }}{{n_{i} - n_{i - 1} }}$. The clusters are sorted so that the relative risks of cluster *A*_*i*_’s are non-increasing. Note that

2 $$ c_{i} \ge n_{i} $$

and

3 $$ \frac{{c_{i} - c_{i - 1} }}{{n_{i} - n_{i - 1} }} \ge \frac{{c_{i + 1} - c_{i} }}{{n_{i + 1} - n_{i} }} $$

By definition, the Gini coefficient is two times of the shaded area, and can be expressed as

4 $$ G = 1 - \sum\limits_{i = 1}^{I + 1} {(n_{i - 1} + n_{i} )} (c_{i} - c_{i - 1} ) $$

where $c_{0} = n_{0} = 0$ and $c_{I + 1} = n_{I + 1} = 1$. With some algebra, Gini coefficient can also be expressed as

5 $$ G = \sum\limits_{i = 1}^{I + 1} {(n_{i} c_{i - 1} - n_{i - 1} c_{i} )} $$

### Theoretical features of Gini coefficient

The value of the Gini coefficient is between 0 and 1, with higher values indicating higher disparity in the clusters. In selecting clusters to report, the cluster set with the highest Gini coefficient value is picked. A nice property of the Gini coefficient is that it is unchanged if the population in all locations are multiplied by the same constant. There are four other important theoretical features that we think that any reasonable selection criterion should have, and we show that the Gini coefficient satisfies all of them. The four features can be expressed as: (1) If two sets of clusters have the same number of clusters and same expected number of cases, the set with more cases should be selected; (2) If two sets of clusters have the same number of clusters, and one set has more cases and expected cases than the other and the excess number is the same for both cases and expected cases, then one should select the cluster set with fewer expected cases; (3) If one set of clusters contain all the clusters in the other set, then the set with more clusters should be selected; and (4) If one set has fewer clusters than the other but the total number of cases and expected cases are the same for the two clusters, the set with fewer clusters should be selected.

To prove that Gini coefficient has all the four features, the following notation is used. Two sets of clusters, set 1 and set 2, are being considered, with the number of clusters being $I_{1}$ and $I_{2}$, respectively. In each cluster *i*, the number of expected cases are *n*_*i*,1_ and *n*_*i*,2_, and the number of observed cases are *c*_*i*,1_ > *n*_*i*,1_ and *c*_i,2_ > *n*_*i*,2_. The Gini coefficients are $G_{1}$ and $G_{2}$ for cluster set 1 and set 2 respectively. Here we will show that the Gini coefficient satisfies the following theoretical criteria.

#### **Theorem 1**

*IfI*_1_ = *I*_2_ = *I*, *n*_*i*,1_ = *n*_*i*,2_ = *n*_*i*_*for alli*, *c*_*i,*1_ ≥ *c*_*i*,2_*for alli*, *andc*_*i*,1_ > *c*_*i*,2_*for at least onei*, *then*$G_{1} > G_{2}$*. This criterion guarantees that when comparing two sets of clusters with the same number of clusters and the same expected counts, the set with uniformly more cases is selected to be reported*.

#### *Proof*

It can be shown that the difference in the Gini coefficients for the two sets of clusters, *G*_1_ and *G*_2_, is calculated as

$$ \begin{aligned} G_{1} - G_{2} & = \sum\limits_{i = 1}^{I + 1} {\left[ {(n_{i,1} c_{i - 1,1} - n_{i - 1,1} c_{i,1} ) - (n_{i,2} c_{i - 1,2} - n_{i - 1,2} c_{i,2} )} \right]} \\ & = \sum\limits_{i = 1}^{I + 1} {\left[ {(n_{i} c_{i - 1,1} - n_{i - 1} c_{i,1} ) - (n_{i} c_{i - 1,2} - n_{i - 1} c_{i,2} )} \right]} \\ & = \sum\limits_{i = 1}^{I + 1} {\left[ {(n_{i} (c_{i - 1,1} - c_{i - 1,2} ) - n_{i - 1} (c_{i,1} - c_{i,2} )} \right]} > 0 \\ \end{aligned} $$

Using similar algebra, we can prove the following features are also true for Gini coefficients.

#### **Theorem 2**

*IfI*_1_ = *I*_2_, *n*_*i*,2_ = *n*_*i*,1_ + *kandc*_*i*,2_ = *c*_*i*,1_ + *kfor alli*, *andk* ≥ 0, *then*$G_{1} \ge G_{2}$. *This feature means that when comparing two sets of clusters with the same excess count* (*c*_*i*_ − *n*_*i*_), *the selection rule will favour the set with the smaller expected counts*.

#### **Theorem 3**

*If I*_1_ > *I*_2_, $n_{i,j} = n$*and*$c_{i,j} = c$*for all clustersiin both setsj*, *then*$G_{1} > G_{2}$*. This criterion means that if there are multiple identical clusters in the two sets to be considered, the one with more clusters will be picked*.

#### **Theorem 4**

*If*$I_{1} \le I_{2}$, $\sum\nolimits_{i = 1}^{{I_{1} }} {c_{i,1} }$ = $\sum\nolimits_{i = 1}^{{I_{2} }} {c_{i,2} }$*and*$\sum\nolimits_{i = 1}^{{I_{1} }} {n_{i,1} } = \sum\nolimits_{i = 1}^{{I_{2} }} {n_{i,2} }$, *then*$G_{1} \ge G_{2}$*. This criterion states that if there are multiple clusters, and they can be joined into a fewer number of clusters without adding anything else, then the set with the joined clusters is preferred*.

### Data and simulation examples

In order to evaluate the performance of the Gini coefficient, we use both actual cancer mortality data and simulated data on selected cancer sites. To gain a complete understanding of the performance of the proposed criteria, we select cancer sites of various levels of frequency. For rare cancers, the total number of deaths in a single year will be too small to produce reliable estimates of relative risks, so multiple years of data are aggregated to mitigate this problem. Table 2 lists the selected cancer sites, the total number of deaths for these cancers in year 2006, and the time span the mortality data are aggregated. In addition to the actual cancer mortality data, for each cancer site, simulated data are created to evaluate the proposed criterion. The simulated cancer mortality data are created based on the Gini chosen clusters for the actual mortality data, with the same level of relative risks for the clusters.

**Table 2:** Cancer sites of the actual US cancer mortality data, 2006

Cancer site	Total number of deaths	Years of data aggregation
Lung, male	88,791	2006
Lung, female	69,037	2006
Breast	40,600	2006
Prostate	28,256	2006
Ovary	14,781	2002–2006
Bladder, male	9368	2000–2006
Bladder, female	4049	2000–2006
Cervical	3953	2000–2006

The other simulated data are from the Northeastern USA benchmark data sets published on the SaTScan website www.satscan.org/datasets.html and described in detail before [15]. We use several simulated data sets from this source with both 600 and 6000 cases. Three different sets of local clusters are constructed in a rural, urban, and mixed urban/rural area respectively. Within each of these three sets, cluster sizes vary with 1, 2, 4, 8, and 16 counties respectively. The data sets *rural*, *urban* and *mixed* contain single hot-spot clusters around Grand Isle, New York City, and Pittsburgh respectively.

**Fig. 4:**
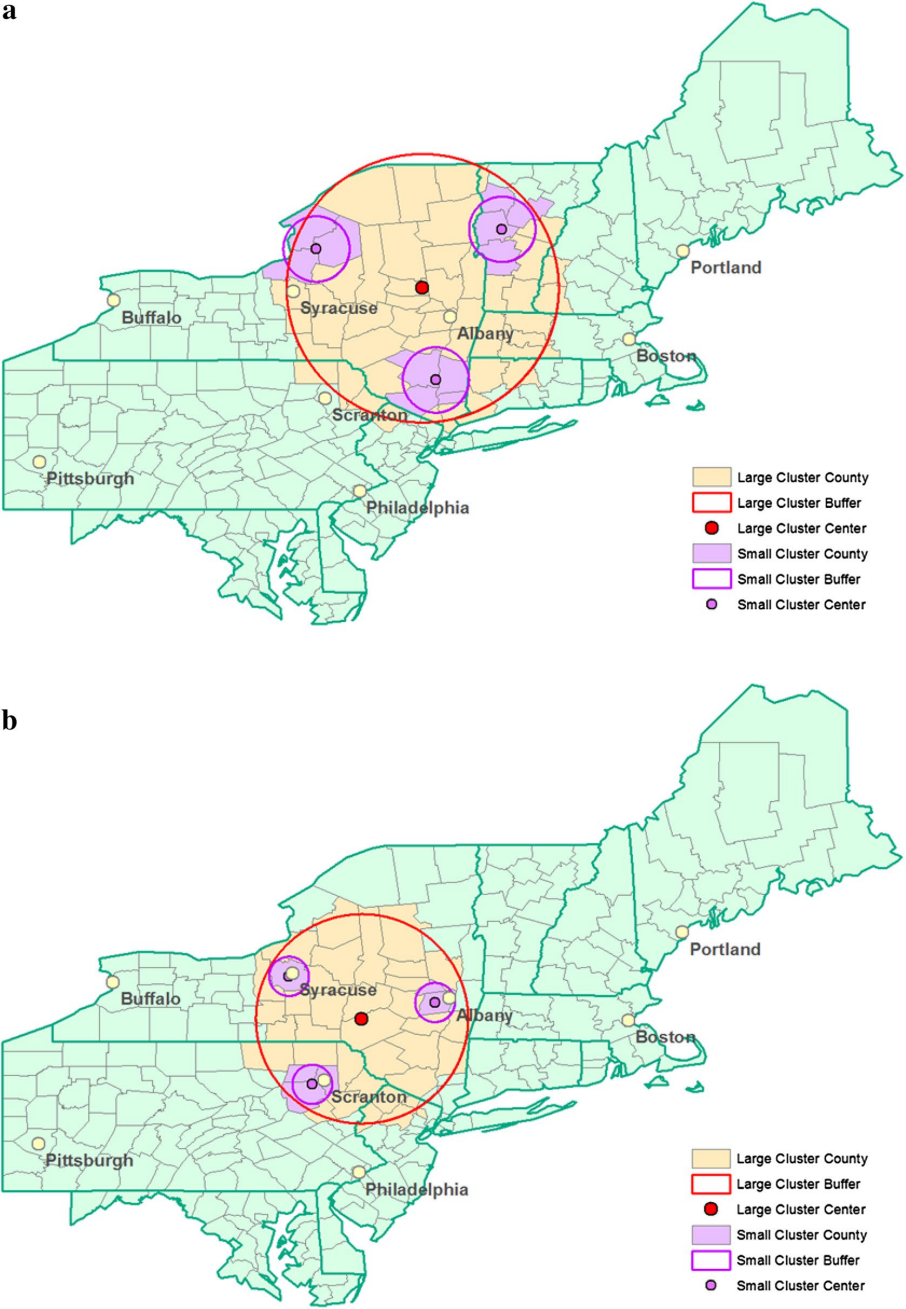
Simulated cluster configurations with one large cluster and three smaller clusters. **a** Urban center with small rural clusters. **b** Rural center with small rural clusters

In situations that one big cluster or several small clusters exist in a study area, the hierarchical way of reporting clusters in SaTScan will usually detect one big cluster in both situations when 50 % is set as the maximum spatial window size. The Gini coefficient may perform better and settle on one big cluster in the first case and a few smaller ones in the other. To evaluate this, the third simulated data is created using the same geographical area as the Northeastern USA benchmark data. Large and small clusters are created in the same area, as shown in Fig. 4. Configuration A (urban center with small rural clusters) consists of a large cluster of counties with an urban area in its center (Albany, NY) and three smaller clusters within the same area that consist of rural counties. Configuration B (rural center with small urban clusters) consists of a large cluster of counties with a rural area in its center and three smaller clusters within the same area that consist of urban counties (Albany, NY, Syracuse, NY, and Scranton, PA).

## Results

Table 3 shows the optimal maximum reported cluster size (MRCS) chosen based on the Gini coefficient for both actual and simulated cancer mortality data. The clusters reported with either the best MRCS, or the second best MRCS when a 50 % MRCS is identified as the optimal, are used to create the simulated data. The second best MRCS is shown in parentheses under the “Actual Data” column. With the total number of cases fixed as in Table 2, we generate simulated datasets for each of the eight cancer sites. For male lung cancer mortality, Gini reports 50 % as the optimal MRCS, and the second best MRCS is of size 10 %. The simulated male lung cancer mortality data is then created using the cluster model reported at the 10 % MRCS level. Gini coefficient correctly identifies 10 % as the optimal MRCS for the simulated data. For the actual female lung cancer mortality data in 2006, Gini coefficient picks 15 % as the optimal MRCS. In the simulated female lung cancer mortality data, Gini coefficient correctly identifies 15 % as the optimal MRCS as well. Actually, the Gini coefficient always identifies the correct MRCS for all the eight simulated data in Table 3. For actual female lung cancer mortality data in 2006, Fig. 5 plots the value of Gini coefficient at the various MRCS levels. MRCS with the highest Gini value, at 15 % of population, is the optimal cluster reporting size.

**Table 3:** Optimal maximum reported cluster size (MRCS, in percent of population) chosen by the Gini coefficient for actual cancer mortality data and simulated cancer mortality data

Cancer site	Year(s)	Actual data^a^	Simulated data
Male lung	2006	50 (10)	10
Female lung	2006	15 (10)	15
Breast	2006	30 (25)	30
Prostate	2006	10 (2)	10
Ovary	2002–2006	25 (30)	25
Male bladder	2000–2006	5 (10)	5
Female bladder	2000–2006	50 (15)	15
Cervical	2000–2006	5 (10)	5

^a^Numbers in parentheses are the second best MRCS chosen by Gini coefficient

**Fig. 5:**
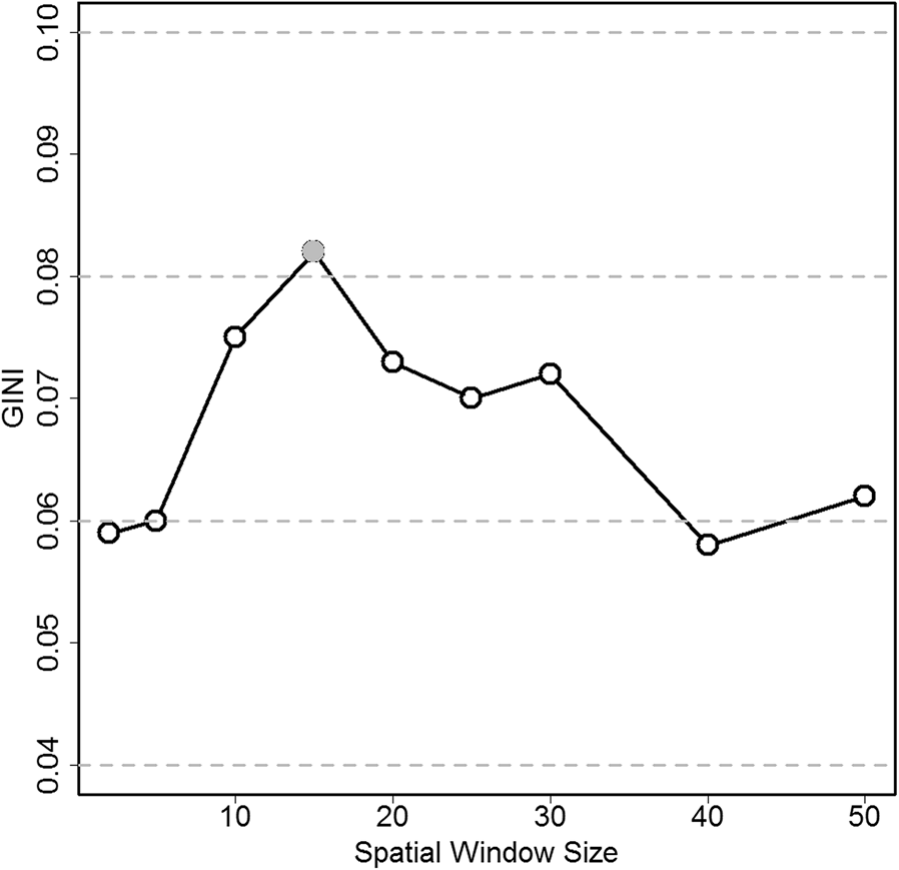
Values of Gini and CLIC at various maximum spatial window sizes for U.S. Female Lung Cancer Mortality, 2006

Another simulation study is based on the Northeastern USA benchmark data sets. Data sets *rural*, *urban*, and *mixed* contain one cluster in a rural, urban, or mixed urban/rural area, respectively. Table 4 shows the optimal MRCS determined by the Gini coefficient values using the Northeastern USA Benchmark data with either 600 or 6000 total cases. As shown in the table, as the number of counties in the cluster increases, the percent of population in the “true” cluster increases, and the MRCS picked by the Gini coefficient varies, depending on the location of the cluster. If the true cluster is located in a rural area, then the MRCS required to pick the right cluster is 1 or 2 % in most cases, because the percentage of population in the true cluster is always below 2 %. If the true cluster is in an urban area, then as the number of counties increase in the cluster, so does the population in the cluster (from 2.7 to 25.8 % of all the population in the study area). The optimal MRCS increases from 3 to 40 % in the scenario of 600 cases and from 3 to 30 % in the scenario of 6000 cases. If the true cluster is located in a mix of rural and urban areas, the optimal MRCS remains in the level close to the percent of population in the true cluster. In the case of two or multiple clusters (data not shown), there is not much difference in the MRCS as the number of counties increase in the cluster.

**Table 4:** Optimal MCRS identified by the Gini coefficient in the Northeastern USA benchmark data

# counties	% pop in cluster	Optimal MRCS (%)	
		600 cases	6000 cases
Rural			
1	0.01	1	1
4	0.5	1	1
8	0.7	1	2
16	1.2	1	2
Urban			
1	2.7	3	3
4	3.6	10	10
8	10.0	20	25
16	25.8	40	30
Mixed			
1	2.4	6	3
4	2.8	5	5
8	3.8	6	6
16	5.7	6	6

In the third simulation study, we examine the behavior of the Gini coefficient when a large cluster or three small clusters exist in the same regions. The hierarchical way of reporting clusters in SaTScan will more likely report the single large cluster in both situations, while the Gini coefficient can identify the correct large or small clusters more often. Table 5 presents the details of the simulated data, which includes number of counties, the population size, percent of population over the study area, and relative risk in each of the large or small clusters in the two configurations described in Fig. 4. The relative risks are calculated using the algorithm in Kulldorff [15] to guarantee a statistical power of 0.999 under the alternative hypothesis of significant disease clusters. A total of 1000 replications of each configuration are created. Table 5 shows that for the Gini coefficient and the SaTScan hierarchical approach, what percent of the 1000 replications report 1, 2, 3, or 4+ clusters in each of the “One Large Cluster” or “Three Small clusters” scenarios. A better criterion will show higher percentage for 1 cluster in the “One Large Cluster” columns and 3 clusters in the “Three Small clusters” columns. If only one large cluster exists, in both urban center and rural center scenarios, the SaTScan hierarchical approach always identifies 1 large cluster, while Gini detects 1 large cluster 90 % of the time in the urban center and only 76 % of the time in the rural center. When 3 small clusters exist, SaTScan correctly detects 3 small clusters 46 % of the time in the urban center and only 11 % of the time in the rural center scenarios. Gini coefficient outperforms the hierarchical approach with 85 and 56 % correct reporting respectively. While the hierarchical approach does slightly better when there is one large cluster, the Gini coefficient does a lot better when there are many small clusters. Generally, Gini coefficient tends to report smaller multiple clusters while the hierarchical approach tends to report fewer and larger clusters.

**Table 5:** Comparison of the Gini coefficient and the hierarchical cluster reporting criteria using the simulated cluster configurations in the Northeastern USA

	(A) Urban centre with small rural clusters		(B) Rural centre with small urban clusters	
	One large cluster	Three small clusters	One large cluster	Three small clusters
# counties	57	11	40	6
Total population in clusters (%)	4,344,150 (14.7)	681,984 (2.3)	2,736,674 (9.3)	780,451 (2.6)
Relative risk	1.18	1.46	1.23	1.43
Gini				
1	90 %	0	76 %	15 %
2	1 %	12 %	13 %	28 %
3	0	85 %	4 %	56 %
4+	9 %	2 %	7 %	1 %
Hierarchical				
1	100 %	2 %	100 %	64 %
2	0	52 %	0	25 %
3	0	46 %	0	11 %
4+	0	0	0	0

## Discussion

The spatial scan statistic will almost always find multiple overlapping statistically significant clusters, and it is not useful to report all of them. Some users have utilized multiple different maximum spatial window sizes (MSWS) in an attempt to find most important clusters, but that is invalid from a statistical perspective since it does not appropriately account for the multiple testing. Keeping the MSWS fixed, it is valid to try different maximum reported cluster sizes (MRCS), but there has not been an objective criterion for deciding which collection of clusters to present. In practice, the MRCS is either determined arbitrarily in an ad-hoc manner or set at 50 % of the population. We found that setting MRCS at 50 % often results in unnecessarily large and less informative clusters.

## Conclusions

We propose to use the Gini coefficient as a more intuitive and systematic way to determine the best collection of clusters to report. It is not intended to evaluate the statistical significance of disease clusters; instead, it is developed to select a suitable and informative collection of non-overlapping cluster to report, among the many overlapping clusters identified by the spatial scan statistics. Hence, instead of replacing the spatial scan statistics, Gini coefficient enhances the spatial scan statistics by providing a criterion to select the collection of non-overlapping clusters to report. It identified the correct clusters in simulation studies and performed better than the hierarchical cluster reporting option. It also has the important property of being invariant under a uniform multiplication of the population numbers by the same constant. Gini coefficient is also shown to satisfy other important theoretical features.

The Gini coefficient has been implemented in the free SaTScan™ software version 9.3 (www.satscan.org).

## Abbreviations

MRCS: maximum reported cluster size
MSWS: maximum spatial window size
